# Cortical Decoding of Individual Finger Group Motions Using ReFIT Kalman Filter

**DOI:** 10.3389/fnins.2018.00751

**Published:** 2018-11-05

**Authors:** Alex K. Vaskov, Zachary T. Irwin, Samuel R. Nason, Philip P. Vu, Chrono S. Nu, Autumn J. Bullard, Mackenna Hill, Naia North, Parag G. Patil, Cynthia A. Chestek

**Affiliations:** ^1^Robotics Graduate Program, University of Michigan, Ann Arbor, MI, United States; ^2^Department of Biomedical Engineering, University of Michigan, Ann Arbor, MI, United States; ^3^Department of Neurology, University of Alabama, Birmingham, AL, United States; ^4^Department of Biomedical Engineering, Duke University, Durham, NC, United States; ^5^Mechanical Engineering Department, University of Michigan, Ann Arbor, MI, United States; ^6^Department of Neurosurgery, University of Michigan, Ann Arbor, MI, United States; ^7^Department of Neurology, University of Michigan, Ann Arbor, MI, United States; ^8^Neuroscience Graduate Program, University of Michigan, Ann Arbor, MI, United States; ^9^Department of Electrical Engineering and Computer Science, University of Michigan, Ann Arbor, MI, United States

**Keywords:** brain-machine interface, neural prosthetics, cortical decoding, motor control, Kalman filter, individual finger, intention estimation

## Abstract

**Objective:** To date, many brain-machine interface (BMI) studies have developed decoding algorithms for neuroprostheses that provide users with precise control of upper arm reaches with some limited grasping capabilities. However, comparatively few have focused on quantifying the performance of precise finger control. Here we expand upon this work by investigating online control of individual finger groups.

**Approach:** We have developed a novel training manipulandum for non-human primate (NHP) studies to isolate the movements of two specific finger groups: index and middle-ring-pinkie (MRP) fingers. We use this device in combination with the ReFIT (Recalibrated Feedback Intention-Trained) Kalman filter to decode the position of each finger group during a single degree of freedom task in two rhesus macaques with Utah arrays in motor cortex. The ReFIT Kalman filter uses a two-stage training approach that improves online control of upper arm tasks with substantial reductions in orbiting time, thus making it a logical first choice for precise finger control.

**Results:** Both animals were able to reliably acquire fingertip targets with both index and MRP fingers, which they did in blocks of finger group specific trials. Decoding from motor signals online, the ReFIT Kalman filter reliably outperformed the standard Kalman filter, measured by bit rate, across all tested finger groups and movements by 31.0 and 35.2%. These decoders were robust when the manipulandum was removed during online control. While index finger movements and middle-ring-pinkie finger movements could be differentiated from each other with 81.7% accuracy across both subjects, the linear Kalman filter was not sufficient for decoding both finger groups together due to significant unwanted movement in the stationary finger, potentially due to co-contraction.

**Significance:** To our knowledge, this is the first systematic and biomimetic separation of digits for continuous online decoding in a NHP as well as the first demonstration of the ReFIT Kalman filter improving the performance of precise finger decoding. These results suggest that novel nonlinear approaches, apparently not necessary for center out reaches or gross hand motions, may be necessary to achieve independent and precise control of individual fingers.

## 1. Introduction

Recent clinical trials have demonstrated the use of neural prostheses to restore motor activity in individuals with severe paralysis (Wodlinger et al., 2015; Willett et al., 2017). Brain machine interfaces (BMIs) provide intuitive control signals that are particularly useful for focused upper-limb movements such as reaching tasks and tool use. These signals can last for years (Simeral et al., 2011; Gilja et al., 2012b; Hochberg et al., 2012; Collinger et al., 2013) enabling, at minimum, long term feasibility studies. They are a natural choice for improving the control of functional electrical stimulation (FES) systems, which apply small electric pulses to muscles to produce movement. Indeed, restoring natural movement of the arm is more desirable to people with cervical level SCI than controlling a robotic prosthesis (Blabe et al., 2015). With existing FES systems, certain patients with spinal cord injuries can regain partial use of their paretic hands controlled via residual muscle signals (Kilgore et al., 2008). More recently, this has been demonstrated using BMIs, restoring a small number of functional movements during activities of daily living (Bouton et al., 2016; Ajiboye et al., 2017). These work demonstrate the immense progress that has been made in the field. However, performance limitations still persist, including the inability to activate joints simultaneously or command complex grasps.

Early studies demonstrated neural decoders, which translate neural signals from the motor cortex into motion commands, successfully gave NHPs online control of a robotic arm capable of whole arm movements and a basic grasp (Taylor et al., 2002; Carmena et al., 2003; Serruya et al., 2003; Lebedev et al., 2005; Velliste et al., 2008). While these studies represented major advances, they involved relatively rudimentary movements. Since these work, advances in experimental setups, implantation hardware and techniques, signal processing and control algorithm development have positioned the community to investigate whether decoders can account for more complex multi-joint movements. More recent clinical studies have demonstrated online control of a robotic prostheses in human patients with tetraplegia (Hochberg et al., 2012), with increasing degrees of freedom (DOF) (Collinger et al., 2013), and the ability to activate different grasp patterns(Wodlinger et al., 2015). The selection of multiple grasps has also been demonstrated in recent FES systems (Colachis et al., 2018). Although these studies are impressive demonstrations in a practical setting, grasps were limited to a simple open-close, a few gross motions, or discrete selections. In general, precision typically decreases as subjects are given control of more DOF. Neural prostheses will ultimately need to provide more dexterous hand functionality to allow users to fully interact with the world around them to gain broad acceptance. Newer upper-limb decoders continue to promise increased levels of performance and reliability (Susillo et al., 2016; Shanechi et al., 2017). However, their application has been limited to decoding arm reaches and have yet to demonstrate improvements in grasp performance. Some studies have proposed using semi-autonomous control of a robotic arm to achieve more complex grasps (Downey et al., 2016; Hotson et al., 2016). While this may be a well-received solution for some users, it hands off a majority of fine motor control to an autonomous system, thereby limiting grasping capabilities to what the chosen algorithm can learn and execute. Here we will focus on direct control strategies, specifically the challenge of decoding control signals for precise hand motions for eventual use in an FES system.

The human upper-limb is a high dimensional system: arm and wrist joints provide 7 DOF while the hand is a complex 23 DOF end-effector. Previous NHP studies have shown neural signals can be used to reconstruct 18 (Aggarwal et al., 2013), 25 (Vargas-Irwin et al., 2010), and even 27 (Menz et al., 2015) DOF offline during reach and grasp movements. However, these studies only demonstrate that DOF of the hand are well correlated with primary motor cortex (M1) activity during highly coordinated grasping movements. They do not demonstrate that motion in these DOF can be approximated or controlled individually. Principal component analysis or other forms of dimensionality reduction can be used to characterize the majority of hand motions in fewer well-separated DOF which have been shown to dramatically improve the performance of discrete offline classifiers (Schaffelhofer et al., 2015). Even though it is unclear if the principal dimensions are actually represented in the motor cortex (Mollazadeh et al., 2014), using them for decoding can theoretically provide more precise control over a large amount of hand configurations (Rouse and Schieber, 2015). Recent algorithms have successfully given NHPs the ability to actively select and continuously modulate 4 principal movement dimensions of a virtual hand (Rouse, 2016). Although such techniques have proven to be a promising and efficient way to process information, control is achieved through long periods of reward-based training and is not a reproduction of biomimetic motions. Furthermore, studies have yet to demonstrate precise control along these dimensions.

The body of previous work has successfully demonstrated that information relating to fine motions is well represented in M1. This begs the question if it is possible to use BMIs to control continuous motion at the level of individual fingers. Earlier NHP studies have established that M1 may contain enough information to distinguish between individual finger movements (Hamed et al., 2007; Aggarwal et al., 2008). In our previous NHP work, we characterized the flex-extend motion of all four fingers together as a single DOF, and used signals from M1 to provide subjects with online continuous control of a virtual hand (Irwin et al., 2017). Here we have developed a novel manipulandum to track and control movement of two separate finger groups. Furthermore, we seek to improve decoder performance with the use of the ReFIT (Recalibrated Feedback Intention-Trained) Kalman filter which has proven successful in reach tasks (Gilja et al., 2012b). We use the manipulandum in combination with the ReFIT Kalman filter to provide our NHP subjects with continuous control of each finger group in a separate fashion. To our knowledge, this is the first systematic and biomimetic separation of digits for continuous online decoding in a NHP as well as the first demonstration of the ReFIT Kalman filter improving the performance of precise finger decoding.

## 2. Materials and methods

### 2.1. Novel manipulandum

**Figure 1:**
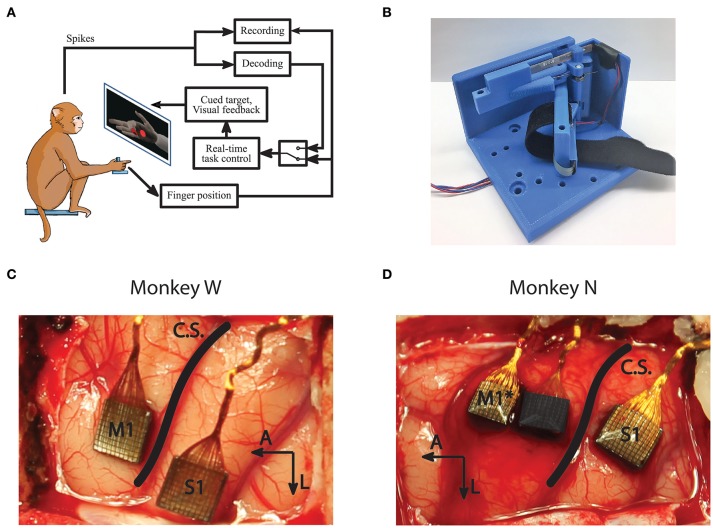
Experiment setup and methods. **(A)** Subjects performed a target acquisition task using a virtual hand controlled either by flex sensors or neural decoder output. **(B)** A novel manipulandum was used to separate finger movements into three active configurations: index only; middle, ring, and pinky (MRP); or all fingers moving together. An unrestrictive stanchion (not pictured) that does not restrict finger movements was also used for sensory context experiments. **(C,D)** Array implants for Monkeys W and N. Only signals from the motor arrays were used in this study. *Monkey N was implanted with a split array, however 64 channels (darkened) were inactive for the study period.

All experimental tasks were performed in compliance with NIH guidelines as well as the University of Michigan's Institutional Animal Care & Use Committee and Unit for Laboratory Animal Medicine. We trained two male rhesus macaques, Monkey W and Monkey N, to use a novel manipulandum, designed to isolate finger movements (Figure 1B), in order to match fingertip position targets in the same virtual environment described in previous work (Irwin et al., 2017). The manipulandum consists of two "doors" with dividers to isolate index finger movements from MRP movements. The doors can be locked separately or together in different positions to create a wide range of movement conditions. Here we used three configurations, each resulting in 1 DOF control:

- Index: MRP door locked to full extension, index finger allowed to move
- MRP: index door locked to full extension, MRP fingers allowed to move
- All: index and MRP doors are locked together to encourage simultaneous movement of all fingers

Switching between configurations can be done in a short period of time, allowing us to alternate between finger groups in a single session. Resistive flex sensors (FS-L-0073-103-ST, Spectra Symbol, Salt Lake City, UT) were attached to each door of the manipulandum and values were read from a 10 bit analog-to-digital converter (ADC) on a custom circuit board. At the beginning of each experiment day, ADC values corresponding to full flexion and extension of the active DOF were noted. The amount of flexion at any given time was then determined by centering and scaling the current ADC value such that a position of 0 corresponded to the recorded value for full extension and 1 corresponded to the value for full flexion. Each door also contained a torsional spring which was tuned to apply as little resistance as possible during flexion, but still allowed the door to follow the subject's fingers during extension.

### 2.2. Behavioral task

Both subjects performed a target matching task in an electrically shielded room with their right forearm flexed 90 degrees, comfortably restrained inside of an acrylic tube. Their hand rested on a table and inserted into the manipulandum. Position data and neural data were acquired in real-time using xPC Target (Mathworks, Natick, MA, United States). The xPC received UDP packets with neural data marking threshold crossings on array channels. The specific thresholding scheme is described below in Signal Processing and Feature Selection. The real-time execution of the xPC ensured that neural and behavioral data were synchronized with millisecond precision. The subjects viewed a virtual hand (MusculoSkeletal Modeling Software; Davoodi et al., 2007), with finger group animations controlled by the xPC to either measured flex sensor data or decoded finger position. The virtual hand was controlled via either the flex sensors for decoder calibration and offline analysis or the predictions from a decoding algorithm for online BMI control (Figure 1A). The experiments for this study only involved control of 1 DOF at any point in time. For future studies, the system is capable of animating trajectories for simultaneous control of multiple DOF.

**Figure 2:**
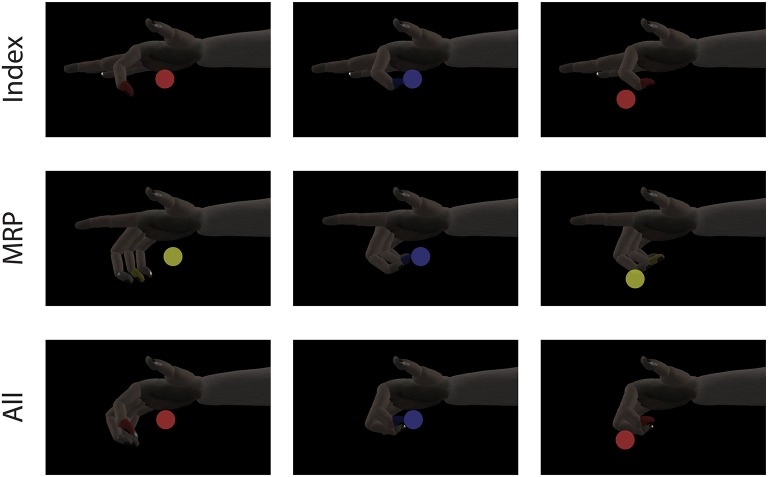
Single DOF target tasks. Each row shows a target sequence for a different finger configuration. For Index and MRP configurations, both the display and manipulandum of the inactive group were fixed to extend. A single trial consists of a target being presented (first column) and the subject moving his active finger group to the target (second column). After the subjects satisfies the required hold time or the trial times out, a new target is presented immediately to begin the next trial (third column).

At the start of each trial, a spherical target is placed along. the flex-extend arc and the subject must move the virtual fingertip inside and remain in the target for a given hold period. The range for a successful hold was considered to be within the visible edges of the spherical target and the required hold period varied between 500 and 750 *ms* depending on the subject and experiment. Successful trials were rewarded with apple juice. Figure 2 shows an example target sequence for each finger configuration. To encourage separation of finger movements, behavioral training was almost always done using either the index or MRP group to acquire center-out targets. Here, “center-out” means that targets appear in 1 of 7 positions along the flex-extend arc with every other target appearing at “center” (50% flexion). At the time of this study, Monkey W was an experienced BMI user, previously trained to move all four fingers together with his hand positioned in a stanchion that did not impede or restrict finger movement in any way. Monkey N was a new user who had only been trained with the manipulandum restricted to index or MRP movements. The first time Monkey N performed the task with the unrestrictive stanchion was during the sensory context experiments described later.

### 2.3. Surgical procedure

The surgical procedure was performed in compliance with NIH guidelines as well as the University of Michigan's Institutional Animal Care & Use Committee and Unit for Laboratory Animal Medicine. We implanted each monkey with intracortical electrode arrays targeting the hand area of left primary motor cortex (M1), as identified by surface landmarks (Figures 1C,D). After the craniotomy, the genu of the arcuate sulcus was identified and a line was traced posteriorly to central sulcus. Arrays were then implanted along this line just anterior to central sulcus, as allowed by vasculature. Both subjects were implanted with Utah arrays (Blackrock Microsystems, Salt Lake City, UT, United States) targeting M1 as well as the left primary sensory cortex (S1). The arrays implanted in both subjects had 1.5 *mm* long electrodes spaced with 400 μ*m* pitch. Monkey W received 96-channel arrays in both M1 and S1, while Monkey N received a 96 channel array in S1 and a 128 channel split array covering M1 with one array potentially overlapping with pre-motor cortex (PMd). Note that only the motor arrays were used for analysis and decoding in this study. Due to wiring damage, Monkey N only had 64 channels of neural input available for this study (Figure 1D). The active array was the one implanted on the rostral edge of the M1 area, so it is possible that units recorded from his array are located in PMd. Similar studies for arm reaches have used signals from both M1 and PMd for continuous decoding (Gilja et al., 2012b).

### 2.4. Signal processing and feature selection

During experiment sessions, neural data was recorded and processed via a Cerebus neural signal processor (Blackrock Microsystems, Salt Lake City, UT, United States). Neural spikes were detected online by a threshold of –4.5 times the RMS voltage on each channel, after applying a 250 *Hz* high-pass filter to the broadband signal. The signals used here were the voltage difference between each channel and a common ground. Software referencing techniques such Common Average Referencing (Ludwig et al., 2009) were not used in this study but could be explored in future work to provide cleaner signals. Broadband data was sampled and recorded at 30 *kHz*, while the thresholded spikes were also recorded for offline analysis and sent to the xPC for real-time decoding. On each experiment day, channel selection for analysis and decoding was done simply by retaining all channels with an average firing rate >1 *spike*/*s* determined by threshold crossings during a training run. Some high-impedance (> 1 *MOhm*) channels repeatedly showed threshold crossings of large disturbances not reflective of neural activity. Therefore, an additional 13 channels for Monkey N and 3 for Monkey W with no visible information content were excluded regardless of whether or not they met the activity cutoff on experiment days. The cutoff threshold was chosen empirically by visually comparing waveforms to impedances. Although daily impedance measurements were taken, the excluded channel list was determined at the start of the study and not updated. While electrode impedances may vary *in-vivo*, the excluded channels almost always remained above the 1 *MOhm* threshold, ranging from 19.03 *to* 23.16 *MOhm* for Monkey W and 0.98 − 23.19 *MOhm* for Monkey N.

For offline analysis and online decoding, we compared the summed threshold crossings in 50 *ms* time bins to the averaged kinematic data recorded from the flex sensor. Previous work demonstrated processing bin widths in the 50 − 100 *ms* range produces an accurate decoder that is sufficiently responsive for online control (Kim et al., 2008). We selected a 50 *ms* bin width empirically in both subjects while they were being familiarized with BMI control, using a standard Kalman filter. In both subjects, we first tested a 100 *ms* bin width and observed online performance as we decreased the bin width. Anecdotally, we found that 50 *ms* produced a more responsive online decoder without a noticeable trade-off in stability and smaller widths performed inconsistently. For offline analysis and parameter estimation, the raw 1 *kHz* flex sensor data was passed through a moving average filter with a 50 *ms* span before being averaged into time bins. Velocity measurements were then computed by taking the difference between successive position bins and scaling according to bin width.

### 2.5. Kalman filter implementation

A linear Kalman filter was used to predict kinematics of the selected finger group during each session. For this study, we predicted the motion of a single DOF at a time. At the beginning of each experiment day, all parameters for the initial Kalman filter were estimated from a training run of approximately 300 center-out trials with a hold time of 750 *ms* scaled to take up 15% of the flex-extend arc. To reduce the effects of sensor noise during parameter fitting, velocities with magnitude below an empirically chosen threshold (0.2*%flex*/*s*) were set to 0. The state vector for the Kalman filter *X*_*t*_ (1) describes the kinematics of the active DOF with an additional offset term to account for baseline firing rate of each of the *n* active channels in *Y*_*t*_ (2).

(1) $$X_{t}={\left[\begin{array}{ccc} pos & vel & 1 \end{array}\right]}^{T}$$

(2) $$Y_{t}={\left[\begin{array}{cccc} y_{1} & y_{2} & \ldots & y_{n} \end{array}\right]}^{T}$$

The Kalman filter state transition model *A* (3) assumes that the finger group's position is explained perfectly by velocity integration, matching our control implementation (7) in which error is only propagated through the velocity term. Velocity estimates are assumed to be contaminated by zero mean Gaussian noise with covariance matrix *W* (4). The velocity damping coefficient *a*_*v,v*_ and variance $\sigma_{v}^{2}$ were determined each day via maximum likelihood estimation using measured hand kinematics from the training run. On average, the estimation yielded *a*_*v, v*_ = 0.855 ± 0.01 for index sessions and *a*_*v,v*_ = 0.869 ± 0.04 (mean ± s.t.d) for MRP sessions. Here *dt* is equal to the 50 *ms* bin width selected for use.

(3) $$\begin{array}{r} X_{t}=AX_{t-1}+w \end{array}$$

(4) $$A=\;\left[\begin{array}{ccc} 1 & dt & 0\\ 0 & a_{v,v} & 0\\ 0 & 0 & 1 \end{array}\right]\text{ and }W=\left[\begin{array}{ccc} 0 & 0 & 0\\ 0 & \sigma_{v}^{2} & 0\\ 0 & 0 & 0 \end{array}\right]$$

**Figure 3:**
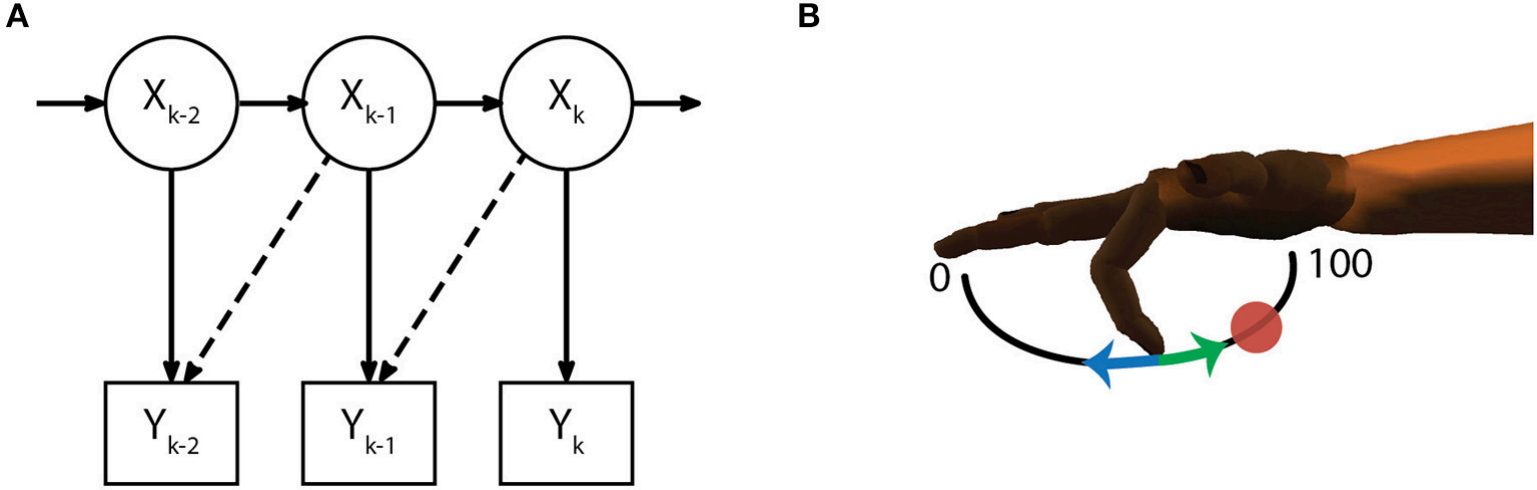
Decoding methods. **(A)** The initial Kalman filter estimates fingertip kinematics based on summed threshold crossings from either the current 50 *ms* time bin or an optimally chosen time lag (dashed lines show 50 *ms* lag). **(B)** Finger motion was characterized in 1 DOF per finger group such that a position of 0 corresponds full extension and 100 corresponds full flexion. Intention estimation was applied to the online kinematics of the initial Kalman filter by taking incorrect decoder velocities (blue arrow) and flipping them (green arrow) to point toward the desired target (red circle), on-target velocities are set to zero. Intention estimation is only applied to estimate parameters for the ReFIT decoder, no knowledge of target locations is used during online control.

The model for the measurement equation (5) assumes a linear relationship between the firing rate of each active channel and each of the kinematic state variables (10), while the additive noise *q* is drawn from a multivariate gaussian distribution with covariance matrix *Q* (6). Parameters for *C* and *Q* were chosen daily via maximum likelihood estimation. Similar to previous work (Irwin et al., 2017), an optimal time lag parameter (0, 1, 2, or 3 bins) was also chosen from the same training dataset (Figure 3A). The time lag is an offset to temporally align bins of neural data with the ideal kinematic measurement. Alternate measurement models (8) and (9) were considered but ultimately discarded (see Measurement Model Selection below).

(5) $$\begin{array}{r} Y_{t-lag}=CX_{t}+q \end{array}$$

(6) $$C=\left[\begin{array}{ccc} c_{1,p} & c_{1,v} & c_{1,o}\\ c_{2,p} & c_{2,v} & c_{2,o}\\ \vdots & \vdots & \vdots \\ c_{n,p} & c_{n,v} & c_{n,o} \end{array}\right]\text{ and }Q=\left[\begin{array}{ccc} \sigma_{1}^{2} & \cdots & \sigma_{1,n}^{2}\\ \vdots & \ddots & \vdots \\ \sigma_{1,n}^{2} & \cdots & \sigma_{n}^{2} \end{array}\right]$$

Drawing from previous work (Gilja et al., 2012b), we used integrated velocity for the virtual fingertip position control signal instead of the predicted position at each time step. Since we assume the subject changes their neural activity in response to both the position and velocity of the virtual fingertip, during online control the position at each time step is set to match the controller output in preparation for the next decode (7).

(7) $$\begin{array}{r} pos_{t|t}=pos_{t-1|t-1}+vel_{t|t}\times dt \end{array}$$

### 2.6. Measurement model selection

Many algorithm implementations have successfully relied on models that only relate neural activity to velocity states (Kim et al., 2008; Collinger et al., 2013; Shanechi et al., 2017). However, neural activity has been observed to also vary with position changes during arm movements, possibly due to differing muscle activation required to maintain postures (Scott and Kalaska, 1997). Previous ReFIT implementations on reach tasks (Gilja et al., 2012b) as well as our earlier Kalman filter implementation for finger motions (Irwin et al., 2017) used a measurement model that included both position and velocity states. Here, we analyzed three choices for kinematic measurement models: position only, velocity only, and position + velocity, shown in Equations 8-10 where *y*_*i*_ is the expected number of threshold crossings in a 50 *ms* time bin for channel *i*.

(8) $$\begin{array}{r} y_{i}=c_{i,p}\times pos+c_{i,o} \end{array}$$

(9) $$\begin{array}{r} y_{i}=c_{i,v}\times vel+c_{i,o} \end{array}$$

(10) $$\begin{array}{r} y_{i}=c_{i,p}\times pos+c_{i,v}\times vel+c_{i,o} \end{array}$$

We performed initial testing with all three measurement models in both subjects after implantation. For finger motions, the position-only and velocity-only models did not provide satisfactory online control, so we opted to continue experiments only with the position + velocity model. We conducted a *post-hoc* analysis to determine the amount of active channels (average firing rate > 1*spike*/*s*) that were “well tuned” to finger activity and showed decent encoding performance with the position+velocity model (cross-validated Pearson's correlation coefficient between predicted and actual firing rate of ρ > 0.05). We also compared the offline decoding performance of the Kalman filters used for decoding days (cross-validated ρ between predicted and actual kinematics) to examine how indicative reconstructions were of our online results.

### 2.7. ReFIT process

The ReFIT Kalman filter was implemented at the beginning of each experiment day using the same two stage process described in previous studies (Gilja et al., 2012a). After performing the task with online brain control with an initial Kalman filter for approximately 200 center-out trials, new measurement model coefficients were determined by regressing recorded neural activity against the intention-estimated online kinematics of the initial decoder (Figure 3B). Intention-estimation alters the training data used for parameter re-estimation by flipping the direction of the predicted velocities to always point toward the target (and setting velocities to zero when the predicted position was inside the target). This process “corrects" velocities with the assumption that the subject always intends to move the virtual hand toward the target, regardless of the direction that was predicted by the initial decoder. The online trials used for retraining were performed with the most challenging center-out targets the subject was able to acquire with the initial Kalman filter. For Monkey W, this was typically smaller targets (15.75% of the flex-extend arc) with longer hold times (750 *ms*), whereas Monkey N required a mixture of reduced hold times (500 *ms*) or larger targets (16.5% of the flex-extend arc). Note that intention estimation was only used during the parameter re-estimation; no knowledge of the target locations was used during online control. Finally, during online processing for the ReFIT filter, we assumed the subjects perfectly internalize the position of the virtual fingertips and adjust their neural activity accordingly. Therefore, the Kalman gain for the ReFIT filter was computed with the assumption of zero *a priori* position uncertainty (11).

(11) $$P_{t|t-1}=\left[\begin{array}{ccc} 0 & 0 & 0\\ 0 & 1 & 0\\ 0 & 0 & 0 \end{array}\right]\left(AP_{t-1|t-1}A^{T}+W\right)\left[\begin{array}{ccc} 0 & 0 & 0\\ 0 & 1 & 0\\ 0 & 0 & 0 \end{array}\right]$$

### 2.8. Individuated finger analysis

Each subject performed one day of experiments to collect offline data to assess the viability of a 2 DOF decoder. On these days, the subject performed the center-out target task (1 *s* hold time, targets take up 16.5% of the flex-extend arc) with hand control of the index or MRP fingers in alternating blocks of approximately 200 trials for 2 sets (approximately 400 total trials per finger group). To determine whether information regarding individuated finger motions was present in M1, we attempted to distinguish between index and MRP movement onset, defined as the 100 *ms* surrounding the time the subject's finger(s) began to flex. Similar to earlier studies that classify individual finger movements (Aggarwal et al., 2008), we used a sliding window which updated every 20 *ms* with the summed threshold crossings of the last 160 *ms* as our neural feature. A support-vector machine with a Gaussian kernel function and *L*_1_ regularization was chosen as the classifier and tested using 10-fold cross validation. A 2 DOF linear Kalman filter was also trained and tested offline across all trials using the same training protocol described earlier. In order to discourage co-contractions in the predicted output, the physical model was constrained to keep each finger group completely independent (13). The measurement model (14) was approximated in a similar manner as the 1DOF case.

(12) $$X={\left[\begin{array}{ccccc} pos_{index} & pos_{mrp} & vel_{index} & vel_{mrp} & 1 \end{array}\right]}^{T}$$

(13) $$A=\left[\begin{array}{ccccc} 1 & 0 & dt & 0 & 0\\ 0 & 1 & 0 & dt & 0\\ 0 & 0 & a_{v,v}^{index} & 0 & 0\\ 0 & 0 & 0 & a_{v,v}^{mrp} & 0\\ 0 & 0 & 0 & 0 & 1 \end{array}\right]\text{ and }W=\left[\begin{array}{ccccc} 0 & 0 & 0 & 0 & 0\\ 0 & 0 & 0 & 0 & 0\\ 0 & 0 & \sigma_{v\;index}^{2} & 0 & 0\\ 0 & 0 & 0 & \sigma_{v\;mrp}^{2} & 0\\ 0 & 0 & 0 & 0 & 0 \end{array}\right]$$

(14) $$\begin{array}{l} \begin{array}{ccc} y_{i} & = & c_{i,pi}\times pos_{index}+c_{i,pm}\times pos_{mrp}+c_{i,vi}\times vel_{index} \end{array}\\ \;\begin{array}{c} +c_{i,vm}\times vel_{mrp}+c_{i,o} \end{array} \end{array}$$

### 2.9. Online experiments

The first group of online experiments was designed to test performance across a variety of target styles and hand configurations. For these, a total of 12 experiments were conducted over 10 days for Monkey W while 8 experiments were conducted over 5 days for Monkey N (Table 1). On each day, subjects were given 1 DOF control over a specific group of fingers (index or MRP). After the ReFIT algorithm was fully trained using center-out targets, the subjects performed the task using online brain control and one of three target styles:

- Center-out (C-O): same as training, targets appear in one of 7 positions along the flex-extend arc with every other target occurring halfway (50% flexion)
- Random (Rand): targets appear in random positions along the flex-extend arc
- Flex-Extend (F-E): targets alternate between flexed (95% flexion) and extended (5% flexion)

**Table 1:** Number of online sessions performed grouped by either finger group or target style.

**Monkey**	**Finger group**			**Target style**		
	**Index**	**MRP**	**All**	**C-O**	**Rand**.	**F-E**
W	6	6	2	6	4	4
N	4	4	2	6	4	-

*Monkey W performed a total of 14 online experiments while Monkey N performed 10. Subjects performed sensory context experiments with “All” four fingers, while target acquisitions sessions were performed with either “Index” and “MRP” finger groups*.

The alternate target styles were chosen to test decoder controllability. The random style is not predictable and requires the decoder navigate untrained trajectories. On the other hand, the flex-extend style is entirely predictable, but exclusively contains difficult targets.

No limits were placed on decoder output so predictions were capable of hyper-extending or over-flexing during online control. To maintain usable feedback during such adverse events a visual limit of −50% to 150% flexion was placed on the virtual fingertips. Otherwise, a disturbance or series of particularly poor predictions could place the virtual hand in state that the subject cannot interpret and correct. The subjects performed the novel task with both the ReFIT and initial Kalman filter in alternating blocks of approximately 50 trials for 3 sets (approximately 150 total trials per decoder). Including the full ReFIT training process and block testing, subjects completed between 826 and 1,252 trials on these experiment days, depending on whether or not multiple target styles were tested. Experiments usually lasted a 2–4 h including time for rig setup and decoder calibration. Monkey W performed two sessions for each of the three target styles with a 750 *ms* hold time and the targets taking up 15.75% of the flex-extend arc. Monkey N performed two sessions of center-out and random target styles with a 500 *ms* hold time and the targets taking up 16.5% of the flex-extend arc.

### 2.10. Sensory context

Previous work has shown that sensory signals from fingertip stimulation are well represented in motor cortex (Schroeder et al., 2017). Although the springs on each door of the manipulandum were selected to provide minimal resistance, the subjects still had the tactile sensation of their fingers pushing on the door. To make sure most of the neural information used by our decoder is primarily a product of motor activity and not a sensory reaction, which may not be a reliable input for SCI patients, subjects performed 2 daily sessions each in which the sensory cue of touching the manipulandum doors was removed. In these sessions, subjects performed these experiments with the manipulandum doors locked together (encouraged to move all fingers together). A ReFIT filter trained with the manipulandum was then used to acquire center-out targets after the manipulandum was replaced with a stanchion that did not interfere with or restrict finger movements, and therefore introduced no additional sensory feedback during movements. Including the initial training period, subjects completed a total of 905–1,063 trials during these experiment days. Performance was compared across decoders and sensory contexts using center-out targets that took up 16.5% of the flex-extend arc and a 500 *ms* hold time.

### 2.11. Online performance metrics

In an attempt to ignore initial adjustments to the decoder, the first 5 BMI trials from each trial block were excluded from performance analysis. To compare performance across experiment sessions while accounting for variations in target difficulties, we used bit rate as our primary performance metric (Thompson et al., 2014). Random trials that required minimal movement to complete (target center appearing within 10% flexion of fingertips) were excluded from this analysis. We did not attempt to draw direct comparisons between the subjects as Monkey W had more experience at the time of these experiments. Furthermore, targets with longer hold times are considerably more difficult to acquire in BMI mode due to orbiting, a phenomena in which the controller oscillates around the desired position and is unable to stop. This increase in difficulty is not fully captured in the metric, despite the hold time itself being excluded from the calculation.

We compared the average acquisition time and average orbiting time for each decoder over all of the center-out decoding sessions. Acquisition time was defined as the time taken to complete a trial from the start to the beginning of a successful hold time. Orbiting time was defined as the time between first target contact and successful acquisition, again excluding the target hold time. Unsuccessful trials are counted as the time between first target contact and the end of the timeout period. Since these metrics can count close to the entire timeout period for unsuccessful trials, they penalize failure more heavily than bit rate. The center-out task was the most conducive for this analysis as it was performed by both subjects and consistently produced targets that were far enough apart to induce orbiting.

## 3. Results

### 3.1. Neural tuning and finger separation

**Figure 4:**
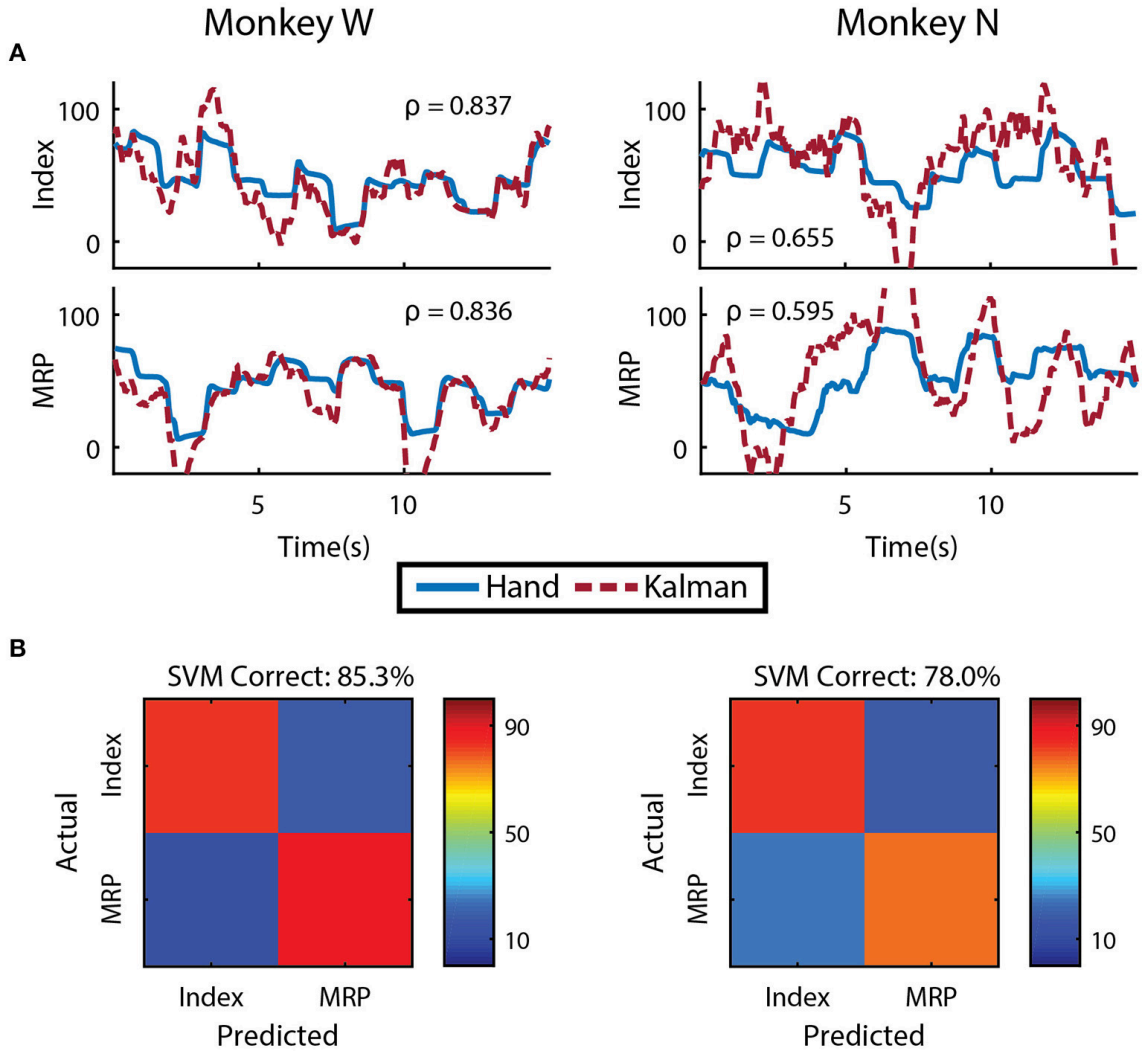
Offline decoding performance. **(A)** Single DOF reconstructions representative of average error on separate training runs for each DOF and subject. Correlations across the entirety of each training run using 5-fold cross validation are shown. **(B)** Cross validated classification of finger group during onset of flex movements using a support-vector machine. Threshold crossings were binned in a sliding window that was updated every 20 *ms*. The feature vector was formed from multiple time bins for all available channels.

We first evaluated whether finger kinematics were well represented in the neural units we recorded. Excluding channels due to low firing rate, artifact activity, or low correlation with finger kinematics as described in the Methods, an average of 48 ± 9 channels from Monkey W and 10 ± 2 channels from Monkey N (mean ± s.t.d) were “well tuned" to finger activity on a given day. Offline Kalman filters from active channels across all training days yielded an average correlation coefficient between predicted and actual position of 0.807 ± 0.074 for Monkey W and 0.655 ± 0.035 for Monkey N (mean ± s.t.d). Example offline decodes are shown in Figure 4A. The average correlation between predicted and actual velocity was 0.618 ± 0.093 for Monkey W and 0.421 ± 0.050 for Monkey N (mean ± s.t.d). Between the two subjects, Monkey W had better offline results and was able to achieve better online performance metrics (see Online Decoding below) while performing a significantly more difficult task (longer hold times, smaller targets) than Monkey N. We suspect that higher quality neural signals, potentially due to a better array location, contributed to Monkey W's better performance. In both subjects, the encoding performance of an active channel was positively (ρ = 0.594 Monkey W and ρ = 0.849 Monkey N) and significantly correlated with its value to the offline decode (Monkey W, *p* < 1 × 10^−9^, *n* = 92 channels; Monkey N, *p* < 1 × 10^−14^, *n* = 52 channels; Student's *t*-test). The value of a specific channel was determined each day by comparing the initial Kalman filter's ability to predict velocity to an offline decoder without that channel (Wahnoun et al., 2006). Across days, we noticed positive but statistically insignificant correlations between offline predictive power and online bit rate, consistent with other studies which note that one is not necessarily indicative of the other (Kim et al., 2008; Ganguly and Carmena, 2010; Cunningham et al., 2011). We also did not uncover any significant correlations between either the quantity of active channels or the number of “well tuned" channels and daily online performance, so we cannot rule out subject motivation and experience as additional performance factors.

To determine if the separate DOFs in our task are well represented in our neural units, we had each subject run an additional offline session in which they alternated between trial blocks of index and MRP target acquisition. Similar to techniques used in Hamed et al. (2007) and Aggarwal et al. (2008), we restricted our analysis to the onset of flex movements from the center position and used a sliding window that was updated every 20 *ms* to including summed threshold crossing over the preceding 160 *ms*. The feature vector used for classification contained all time bins during the movement onset period for every available channel. A support-vector machine, tested with 10-fold cross validation, was able to distinguish between index and MRP flexion with 85.29% accuracy for Monkey W and 78.03% accuracy for Monkey N (Figure 4B). Classification performance suffered when using non-overlapping 20 *ms* bins (71.01% Monkey W and 68.20% Monkey N) or a larger 100 *ms* bin (65.97% Monkey W and 61.98% Monkey N). The poor classification performance with the single 100 *ms* bin suggests that the majority of neurons we recorded were broadly tuned. However supplying the classifier with temporal history at a high resolution appeared to alleviate this issue. Therefore, consistent with previous work (Hamed et al., 2007; Aggarwal et al., 2008), these two finger movements could be distinguished from motor cortex activity.

### 3.2. Online decoding

**Figure 5:**
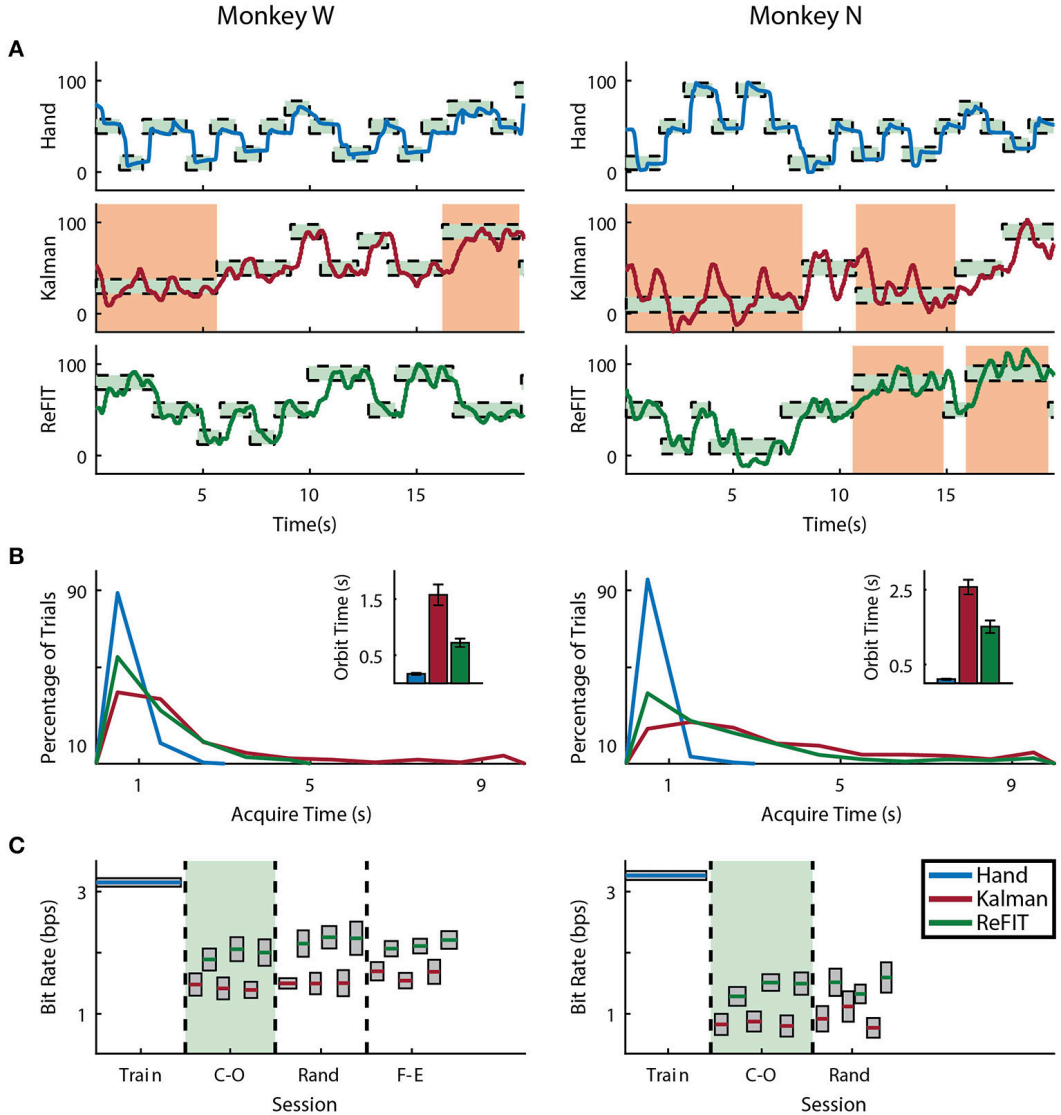
Online index finger decoding performance. **(A)** Traces of the center-out trials closest to session average (session shaded in green below), orange shading indicates trials with > 3 s acquisition time. **(B)** Acquisition times from the same center-out session binned in 1*s* intervals shown along with average orbiting times (mean ± s.e.m.). **(C)** Decoding performance across trial blocks for each target style (mean ± s.e.m.). Dashed lines indicate separate experiment sessions, with a center-out training session shown for a comparison to hand performance. Within each session, the initial Kalman filter trials used to train the ReFIT filter are not shown.

**Figure 6:**
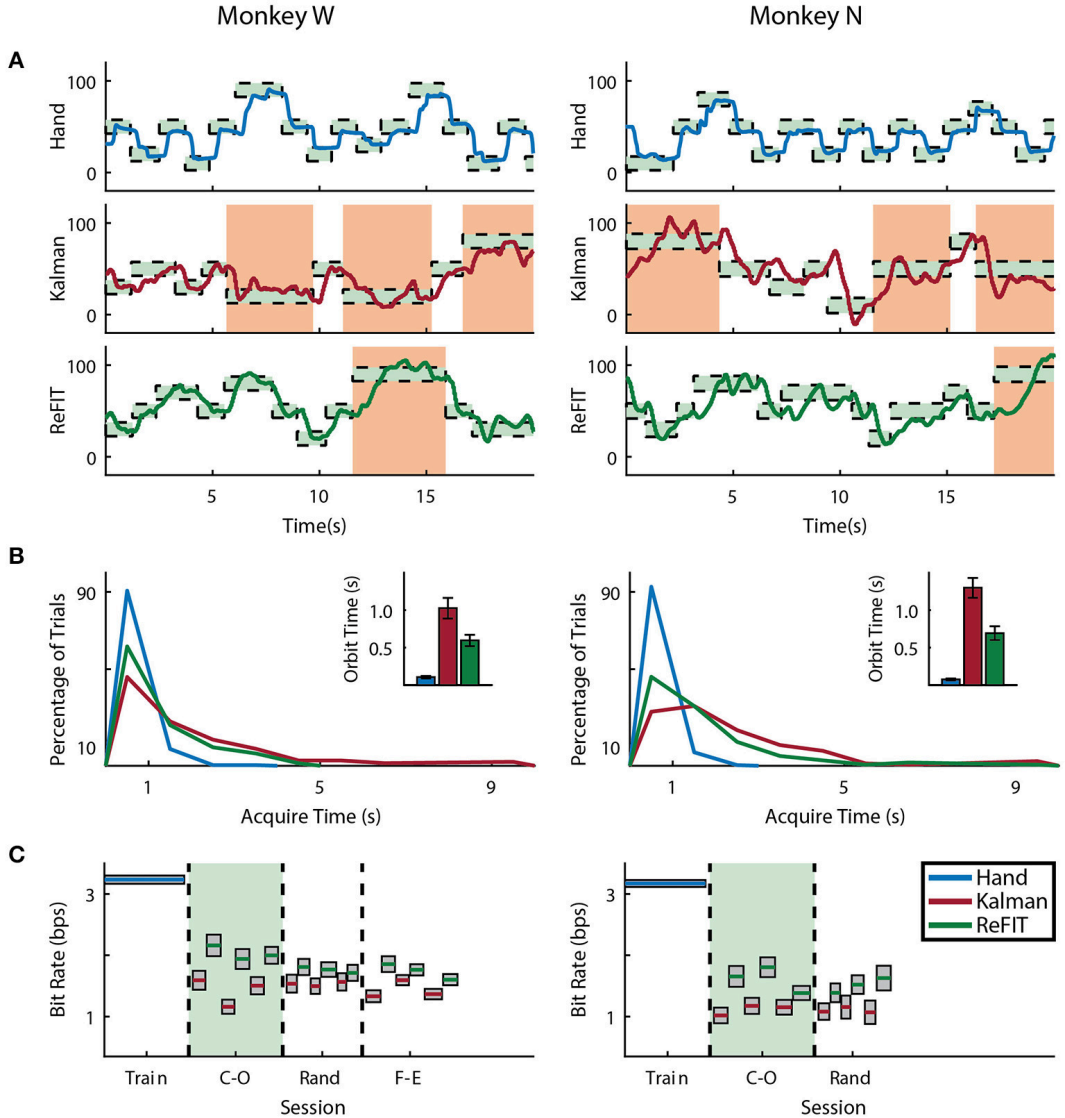
Online MRP finger decoding performance. **(A)** Traces of the center-out trials closest to session average (session shaded in green below), orange shading indicates trials with > 3 s acquisition time. **(B)** Acquisition times from the same center-out session binned in 1*s* intervals shown along with average orbiting times (mean ± s.e.m.). **(C)** Decoding performance across trial blocks for each target style (mean ± s.e.m.). Dashed lines indicate separate experiment sessions, with a center-out training session shown for a comparison to hand performance. Within each session, the initial Kalman filter trials used to train the ReFIT filter are not shown.

Both subjects were able to achieve some level of online control of the virtual hand with the initial Kalman filter as shown in Figures 5A, 6A for index finger and MRP fingers, respectively. Across all sessions, Monkey W achieved an average bit rate with the initial Kalman filter of 1.32 ± 0.29 *bps*, while Monkey N achieved a bit rate of 1.07 ± 0.16 *bps* (bits-per-second; mean ± s.t.d.). The Kalman filter was generally responsive to each subject's input. However, similar to center out literature, sometimes the decoder was unable to stop on-target for the required hold time. Examples of longer trial times, often due to orbiting, are highlighted in orange in Figures 5A, 6A. In some cases, particularly with the initial Kalman filter, orbiting was so severe that the subjects were unable to acquire targets within the timeout period. While using the ReFIT filter, orbiting was generally less frequent and less severe for both index and MRP decoding, resulting in improved performance metrics (example sessions shown in Figures 5B, 6B).

**Figure 7:**
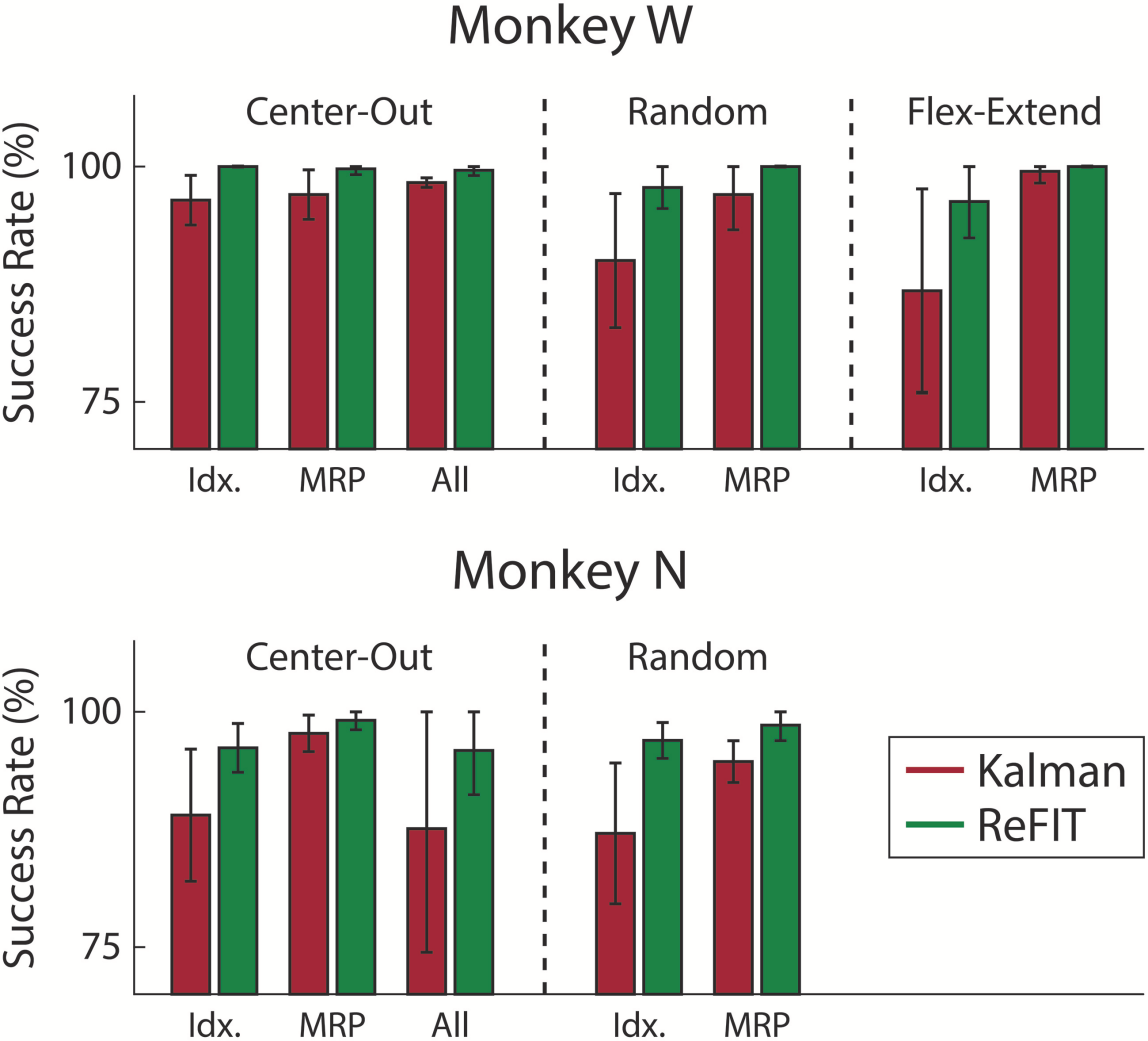
Average success rate of trial blocks across two sessions for each subject (mean ± std). Overall, the ReFIT decoder improved success rate by 4.33 ± 7.19% for Monkey W (*p* < 1 × 10^−5^, *n* = 38 trial blocks; one-sided Wilcoxon sign-rank test) and 5.78 ± 7.35% for Monkey N (*p* < 1 × 10^−4^, *n* = 26 trial blocks; one-sided Wilcoxon sign-rank test).

Consistent with previous studies (Gilja et al., 2012b; Fan et al., 2014), subjects were able to achieve their best online performance with the ReFIT algorithm, which always outperformed the initial Kalman filter throughout the day (Monkey W, *p* < 1 × 10^−7^, *n* = 38 trial blocks; Monkey N, *p* < 1 × 10^−5^, *n* = 26 trial blocks; one-sided Wilcoxon sign-rank test). Example target acquisition sessions for both subjects are shown in Figures 5C, 6C. Figure 7 shows the online success rate averaged across trial blocks for the tasks performed by each subject. Overall, the ReFIT decoder improved success rate by 4.33 ± 7.19% for Monkey W (mean ± s.t.d; *p* < 1 × 10^−4^, *n* = 38 trial blocks; one-sided Wilcoxon sign-rank test) and 5.78 ± 7.35% for Monkey N (mean ± s.t.d; *p* < 1 × 10^−5^, *n* = 26 trial blocks; one-sided Wilcoxon sign-rank test). Across all experiment sessions, the ReFIT algorithm improved the average bit rate by 31.04 ± 2.78% for Monkey W (mean ± s.e.m; *p* < 1 × 10^−36^, *n* = 2114 KF trials and 2010 RF trials; one-sided two sample *t*-test) and 35.17 ± 4.44% for Monkey N (mean ± s.e.m; *p* < 1 × 10^−20^, *n* = 1558 KF trials and 1607 RF trials; one-sided two sample *t*-test). See Supplementary Material for individual session statistics. Furthermore, improvements in bit rate when using the ReFIT decoder were not limited to a particular finger group (Table 2) or target style (Table 3). Overall, while the improvement was robust and reliably present, the increase was not as large as was previously observed in upper limb center out studies (Gilja et al., 2012b).

**Table 2:** ReFIT performance improvements separated by finger group such that “Index” is the improvement to average bit rate (% mean ± s.e.m) across all sessions and target styles performed with the Index finger active.

**Monkey**	**Finger group (% improvement)**		
	**Index**	**MRP**	**All**
W	38.21 ± 5.04^****^	28.97 ± 3.56^****^	18.38 ± 6.76^*^
N	55.60 ± 10.43^****^	33.97 ± 6.07^****^	17.08 ± 7.04^*^

Total number of sessions for each group is shown in Table 1. Stars indicate a significant improvement of ReFIT over the initial Kalman filter (one-sided two sample t-test with

* p < 0.05 and

**** *p < 1 × 10^−6^)*.

**Table 3:** ReFIT performance improvements grouped by target style such that “C-O” is improvement to average bit rate (% mean ± s.e.m) across all sessions and finger groups acquiring center-out targets.

**Monkey**	**Target style (% improvement)**		
	**C-O**	**Rand**	**F-E**
W	35.28 ± 4.33^****^	30.20 ± 6.04^****^	24.92 ± 4.18^****^
N	31.61 ± 4.95^****^	43.74 ± 9.43^****^	-

Total number of sessions of each type is shown in Table 1. Stars indicate a significant improvement of ReFIT over the initial Kalman filter (one-sided two sample t-test with

**** *p < 1 × 10^−6^)*.

**Figure 8:**
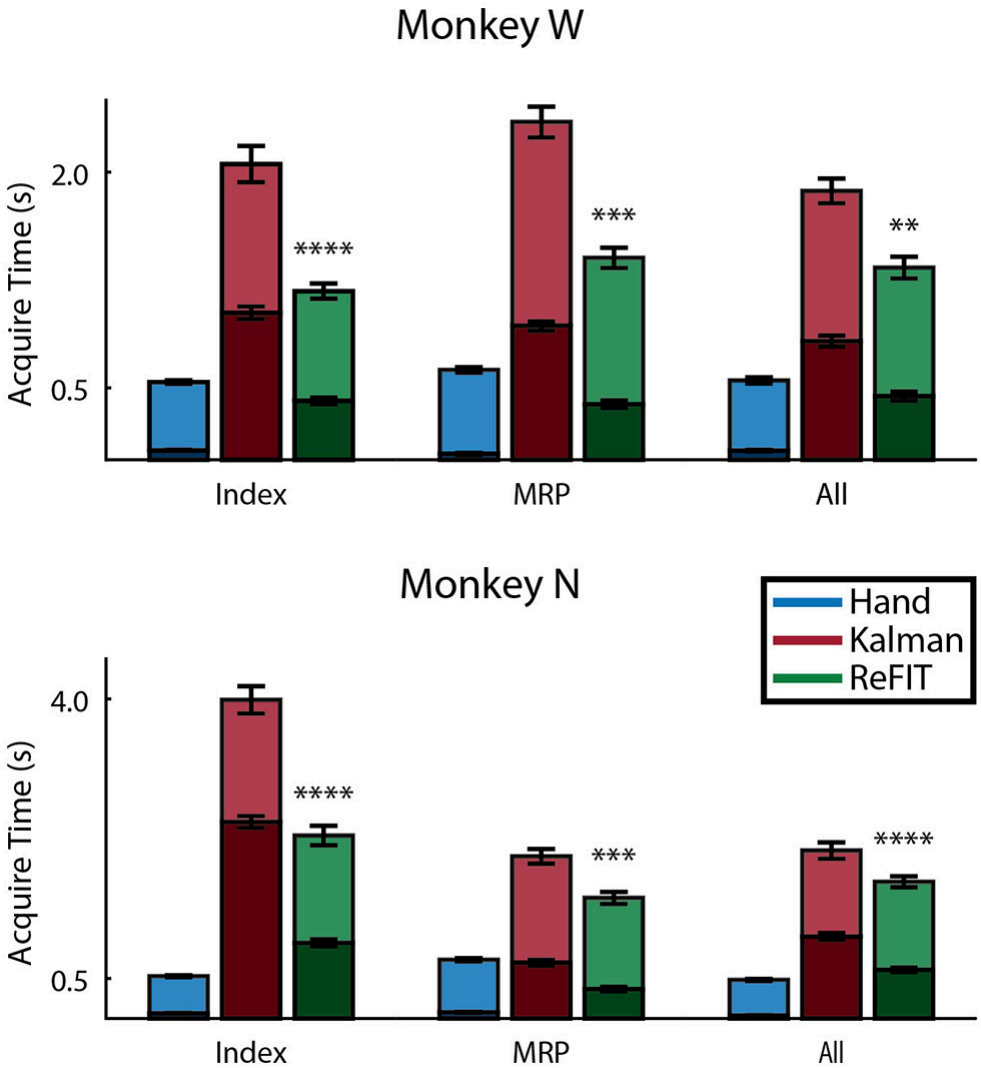
Online performance of center-out target task. Bar graphs indicate average acquisition time (mean ± s.e.m.), with the darkened region scaled to represent the average fraction of each trial spent orbiting (mean ± s.e.m.) across two sessions for each subject. Stars indicate a significant improvement in the orbiting behavior of ReFIT over the initial Kalman filter (one-sided two sample *t*-test, *n* ≈ 300 trials; with ^**^p < 0.001, ^***^p < 1 × 10^−4^, and ^****^p < 1 × 10^−6^).

Figure 8 shows the average acquisition time performing the center-out target task along with the average fraction of each trial spent orbiting. Metrics between both subjects were similar when viewed as percentages. Across all finger groups, using the initial Kalman filter, subjects spent an average of 45.95 ± 0.81% (mean ± s.e.m.) of each trial orbiting the target. Using the ReFIT filter, the average fraction of trial time spent orbiting dropped by 28.21 ± 2.12% (mean ± s.e.m; *p* < 1 × 10^−37^, *n* = 1994 KF trials and 1929 RF trials; one-sided two sample *t*-test). The reduction in orbiting time helped improve average center-out acquisition time by 33.43 ± 2.02% (mean ± s.e.m; *p* < 1 × 10^−37^, *n* = 1994 KF trials and 1929 RF trials; one-sided two sample *t*-test). Consistent with its application in decoding center-out arm reaches (Gilja et al., 2012b), ReFIT significantly increased target acquisition rate primarily due to improved stopping behavior.

### 3.3. Modulation analysis

**Figure 9:**
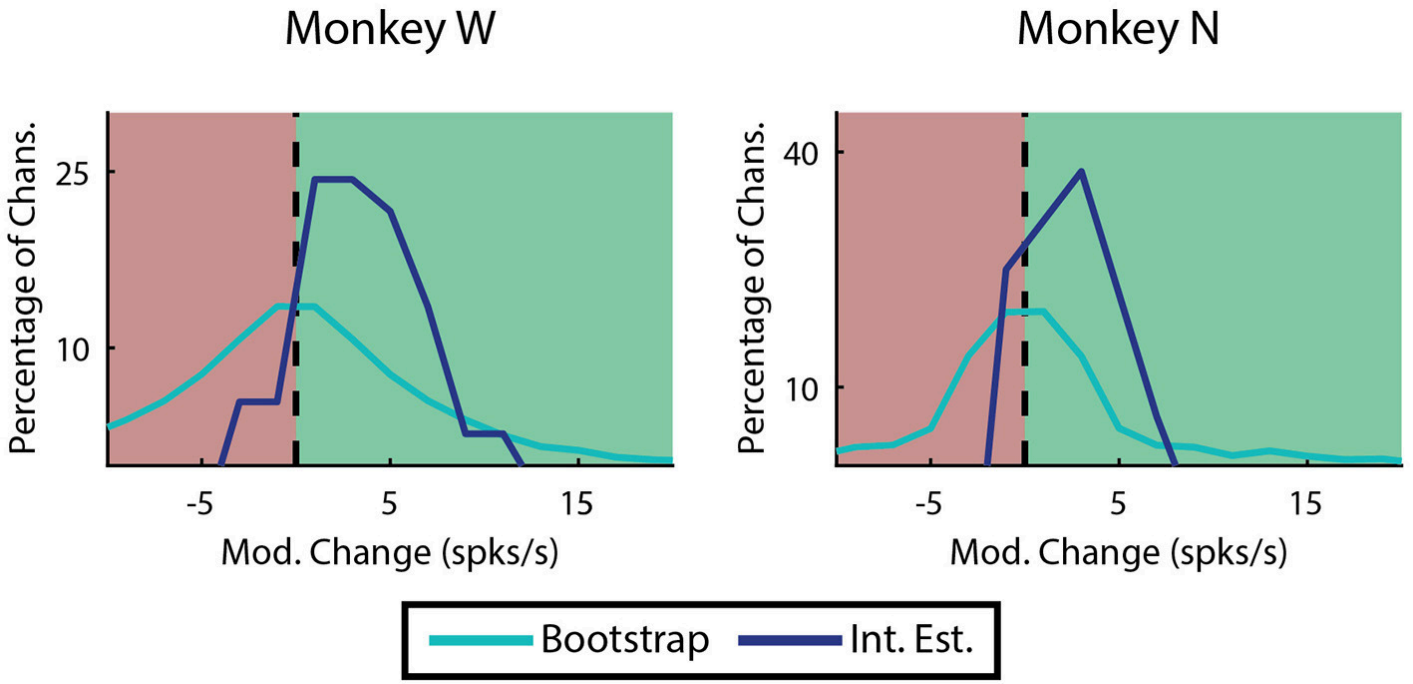
Effects of intention estimation on velocity modulation across all training runs. Analysis was restricted to channels that were originally velocity modulated, had a well correlated linear model, and were valuable to offline decoder performance (*n* = 37 channels over 12 days, Monkey W; *n* = 16 channels over 7 days, Monkey N). The raw change in modulation after intention estimation is applied to the original velocities is shown binned in 2 *spike*/*s* intervals. In both subjects intention estimation improved modulation compared to a randomized bootstrap analysis (*p* < 1 × 10^−7^ Monkey W, *p* < 0.01 Monkey N; one-sided two sample *t*-test).

Most of the improvement of ReFIT for upper-limb movements has been attributed to the sharpening of directional velocity tuning by intention estimation (Fan et al., 2014). Since we characterize finger motions in 1 dimension, velocity tuning is simply represented as the difference in firing rate between flexion and extension. The absolute value of this difference is referred to as modulation. We looked at the change in modulation when intention estimation is applied to the native center-out training data across each monkey's experiment days. Many channels are not well tuned, and therefore are neither expected to benefit or worsen from intention estimation. Therefore, we limited this analysis to “high quality” examples defined as channels on any day that were velocity modulated before applying intention estimation (Mod. >1 *spike*/*s*), had good encoding performance (ρ > 0.1 Monkey W, ρ > 0.05 Monkey N), and were valuable to the decoder. Specifically, the value of a specific channel was determined as described in the Neural Tuning and Finger Separation section above. Here, a negative result indicates the channel is valuable since performance is worse when it is removed. In both subjects, the majority of the high quality channel examples (33 out of 37 channels Monkey W, 12 out of 16 channels Monkey N) chosen for analysis benefited from intention estimation, meaning they showed a raw increase in modulation after intention estimation was applied (Figure 9). Monkey W had an average raw improvement of 3.34 ± 0.51 *spikes*/*s* (mean ± s.e.m.; *p* < 1 × 10^−5^, one-sided Wilcoxon sign-rank test). Monkey N had an average raw improvement of 1.54 ± 0.54 *spikes*/*s* (mean ± s.e.m.; *p* < 0.01, one-sided Wilcoxon sign-rank test). Monkey W had more significant results overall, likely due to the fact that he had more high quality channels available at the time of the study. The positive response to intention estimation suggests that ReFIT may improve performance for finger motions via similar mechanisms observed in upper-limb studies.

### 3.4. Sensory context results

**Figure 10:**
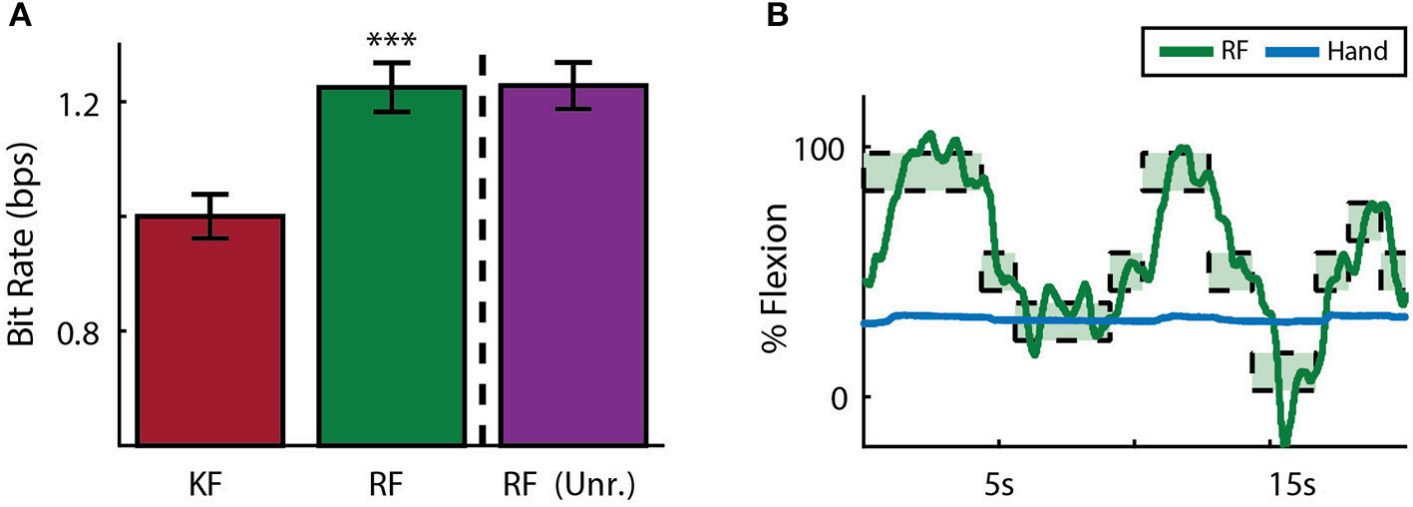
Online decoding under various sensory contexts. **(A)** A ReFIT decoder was trained with the manipulandum to control flexion of all 4 digits. At the dashed line, the manipulandum was removed and subjects continued online control with the same ReFIT decoder (Unr.). Bars represent average bit rate (mean ± s.e.m.) across all sessions for both subjects. For each day and subject, data was normalized by centering such that the average bit rate for the initial Kalman filter was 1 *bps*. Stars indicate a significant improvement of ReFIT over the intial Kalman filter (*p* < 1 × 10^−4^, *n* = 747 ReFIT trials and 781 Kalman filter trials; one-sided two sample *t*-test). **(B)** Target trace of Money W performing center-out target acquisition in BMI mode while voluntarily holding his active MRP fingers still. Green rectangles represent the displayed target ranges the subject successfully acquired.

Each subject also performed two days of sensory context experiments. In these sessions, online performance with the manipulandum moving all fingers was compared to performance after the manipulandum was removed in favor of a stanchion that provided no restrictions on finger movement or tactile finger sensations. To compare performance across subjects, data was normalized across animals by centering around initial Kalman filter performance at 1 *bps*. Across both subjects and sessions, the ReFIT Kalman filter improved performance over the initial Kalman filter (*p* < 1 × 10^−4^, *n* = 747 RF trials and 781 KF trials; one-sided two sample *t*-test). However, there was no statistically significant change using the ReFIT filter across sensory contexts (*p* > 0.9, *n* = 747 manipulandum trials and 780 stanchion trials; two sample *t*-test; Figure 10A). At the minimum, this suggests that the neural information used by the decoders is largely a product of motor activity and subjects are able to adjust to sensory context shifts. Furthermore, we noticed that sometimes subjects will stop moving their active fingers while performing the task in online BMI mode. An example of Monkey W voluntarily holding his MRP fingers still during an online center-target acquisition session is shown in Figure 10B. The subject is still able to control the virtual hand to acquire MRP targets even after electing to hold his MRP fingers still. This indicates that decoding performance is not purely dependent on proprioceptive feedback.

### 3.5. Multiple DOF analysis

**Figure 11:**
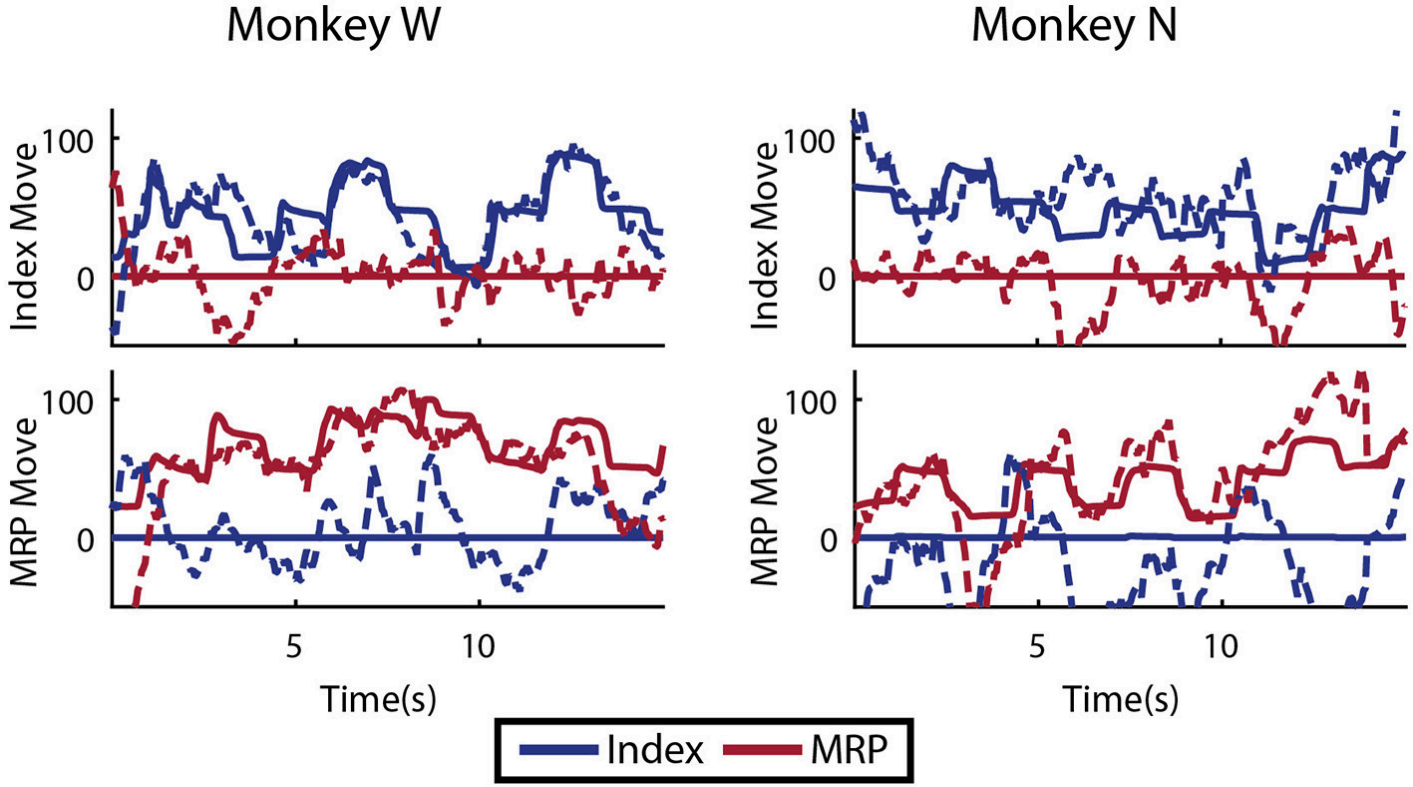
Offline traces of two DOF decoding or both subjects using 5-fold cross validation. Dashed lines are the Kalman filter prediction, while solid lines are the actual finger group position. Training data for this decoder was created by combining multiple training runs, each with a single active DOF. For each subject, intervals where each DOF is active and the other is restricted are shown.

As an initial test of decoding multiple fingers simultaneously, the animals both performed a four block set of alternating datasets (A-B-A-B) for index and MRP fingers. This was decoded offline with cross validation using a Kalman filter state vector that simply included position and velocity for both fingers. As shown in Figure 11, the moving finger had similar decodes to those shown above, however, the motionless finger was usually incorrectly decoded as moving as much or more as the active finger, as shown with example traces in Figure 11. As the physical model (i.e., the *A* matrix) explicitly decouples the fingers, this correlation apparently results from the neural observation model. This is consistent with the modulation depths listed above, in which almost all of the modulated neurons had significant correlations with both index and MRP motion.

## 4. Discussion

Here we showed that NHPs are able to use a ReFIT Kalman filter to acquire fingertip targets using multiple finger groups. The decoder appears controllable in a variety of target configurations and responsive to the subject's intent, as judged by their ability to maneuver to random and unpredictable positions along the finger arcs. Consistent with previous studies, the ReFIT process improved performance over the standard Kalman filter with a substantial improvement in stopping behavior. This was not a given, since the mechanism of improvement is not fully understood, and there is no obvious cosine tuning model (Georgopoulos et al., 1986) for finger movements. However, the performance improvements observed for finger motions are less dramatic than those observed in upper-limb studies (Gilja et al., 2012b). If we consider a purely biomimetic perspective, the increase in modulation via intention estimation shown here and in previous work may result from a reduction in motor noise (Fan et al., 2014). Since fingers are smaller lever arms with actuators designed for precise movements, it stands to reason that there may be less motor noise to eliminate in an able-bodied subject. Secondly, this study and previous work (Irwin et al., 2017) have observed a significant positional tuning element to finger movements. However, our implementation only assumes and corrects noise from velocity measurements. If we look at performance discrepancy from a perspective of decoder adaptation, the task complexity may affect the intention estimation process. The subjects in this study were only given control of 1 DOF at a time: a simple task where a naive decoder has a relatively high chance level of choosing the correct direction at any timepoint. In other words, the chance of false positives with the initial decoder is higher in our 1 DOF task. It is possible that intention estimation techniques may be more impactful when attempting to control multiple degrees of freedom simultaneously, as in a 2 DOF center out task.

In earlier studies, intention estimation was noted as the primary mechanism by which the ReFIT Kalman filter improved performance (Gilja et al., 2012b). The second training stage serves as a closed-loop adaptation period during which the user is presented with visual feedback from the initial Kalman filter and is able to issue corrections in real time which are later processed via intention estimation. Initial decoder parameters may be suboptimal due to poor training data, a shift from physical control to BMI control, or both. Poor training data may be the result of noise in the motor system - discussed earlier, or inconsistent physical behavior. In this context, signal availability and noise from the electrode array is associated with the shift from physical to BMI control. An optimal adaptation to BMI control could be a synergy in which the brain is able to learn a particular decoder and the decoder adapts to better execute commands (Shenoy and Carmena, 2014). Indeed, the brain may identify neurons critical BMI performance and modulate them accordingly (Orsborn et al., 2014). In this view, ReFIT then improves performance by highlighting and better executing commands from these important neurons. In this study and earlier work (Gilja et al., 2012b), a separate re-training step was used, although online learning techniques can potentially update decoder parameters recursively on a shorter timescale (Orsborn et al., 2012; Dangi et al., 2013). Newer adaptive decoders have leveraged more accurate encoding models, improved intention estimation transformations, and assistive training to further optimize parameter convergence (Shanechi et al., 2017). Assistive training strategies combine the output of an initial decoder with an optimal trajectory or enforce constraints along an ideal path during the adaptation phase, typically with decreasing levels of assistance as performance improves. Such techniques incorporate a similar assumption of intention estimation with the addition of online visual feedback of the ideal decoder in a graded fashion. Assistive strategies have successfully provided a paralyzed human subject with high DOF control of a prosthetic arm and hand (Collinger et al., 2013; Wodlinger et al., 2015), and may prove useful as we increase task complexity beyond 1 DOF.

For individual finger control to ultimately be available using neuroprostheses, we must provide control of multiple finger groups simultaneously in an online setting. Decoders that leverage a linear encoding model have been shown to simultaneously control four distinct hand motions in a human subject (Wodlinger et al., 2015). This study is remarkable because it demonstrates that the combination of a well-chosen basis and simple decoder can provide BMI users with modulated control of different grasps in a clinical setting. In the future we aim to provide our NHP subjects with precise online control in a 2 DOF target matching task with the index and MRP finger groups. However, the offline results in this study suggest that linear decoders may not be able to achieve high precision for heavily interdependent finger motions, potentially due to co-contraction. It would be interesting to examine a linear decoder's performance on a subset of the full range of motion we tested. Not only is a reduced range more likely to remain in an area of linearization, but a well chosen range where the subjects can comfortably modulate each DOF with limited natural co-contraction may produce better initial results. It would then be interesting to see if ReFIT or other training techniques allow subjects to generalize to the full range of motion. If the combination of various online linear decoders and intention estimation or adaptation techniques does indeed prove insufficient, there are multiple design directions which may be fruitful. One could develop a more accurate classification scheme to account for and suppress unwanted finger movements, similar to work in reach tasks (Aggarwal et al., 2013; Sachs et al., 2016) and cursor control (Kao et al., 2017). Another study effectively reduced the numbers of DOF of the hand using dimensionality reduction (Rouse, 2016). However, ultimately we need to control multiple fingers at the same time for a hand to be truly useful. We could also seek a more accurate model of the natural muscle synergies that may enable more generalization to multi-finger movements (Nazarpour et al., 2012; Oby et al., 2013; Ethier et al., 2016). These can be incorporated into more general non-linear machine learning approaches, for example including neural networks (Sussillo et al., 2012; Gao et al., 2016). Finally, we could explore contributions from additional information sources (Aflalo et al., 2015) to augment these approaches.

In this study we demonstrated that online finger decoders can be robust to changes in sensory context and proprioception in able-bodied subjects. We hypothesize that had we introduced a context change via increasing spring tension during training, the subjects would be able to compensate with the ReFIT decoder much like they did to other sensorimotor input shifts. As described earlier, the online adaptation of ReFIT may be creating a decoder that is optimal for BMI control of the virtual hand irrespective of existing forward motor path. However, BMIs that promise complex grasps will have to perform under dynamic conditions and disturbances during object manipulation. So while we believe that subjects can compensate for global changes, the rate of environmental adaption may not be sufficient for many realistic conditions. In the future, non-visual feedback mechanisms for BMIs may become available (Flesher et al., 2016), which can greatly improve motor performance provided they are well integrated into the sensorimotor system. Other studies that have examined finger control in able-bodied subjects have observed that the precision of finger control in response to physical disturbances can vary with naturally occurring or induced input noise (Mendez-Balbuena et al., 2012). The authors of that study found that for different individuals introducing a mechanical stochastic noise at different levels produced optimal performance. BMI systems are novel sensorimotor systems where select neurons in the brain interface with either the natural periphery or robotic devices through decoders and encoders. Studying the effects of different types of input noise may be a step toward characterizing and optimizing fine motor control of BMI-FES systems with different feedback modes.

In clinical settings, recent FES systems have provided human patients with the ability to modulate previously paralyzed joints (Bouton et al., 2016; Ajiboye et al., 2017). These implementations show immense potential and represent an amazing convergence of technology and knowledge of the human motor system. As this technology becomes more advanced and transitions further into clinical and ultimately commercial use, further systems level experiments will be required to achieve higher levels of performance in terms of activities of daily living. The everyday functionality of such systems will be limited by both our ability to extract meaningful control signals as well as our ability to actuate multiple joints. It has recently been shown in human subjects that commanding many degrees of freedom simultaneously introduces more error (Ajiboye et al., 2017). In that study, grasp failures with the muscle controller often occurred when the BMI command was generally correct, but small oscillations triggered large swings in other joint movements. While the FES efficacy could be improved with better implantable devices and physical therapy, some of the problem with multiple degrees of freedom will likely require different algorithms. The able-bodied NHP model enables the development of these algorithms with simultaneous knowledge of the complex plant that these neural signals are driving, including significant co-contraction of the muscles. It is not immediately obvious whether the most effective system for individuated finger control will involve directly extracting muscle activation commands from motor cortex or a more agnostic machine learning approach. In a practical setting, the amount of training data required impacts this decision as well, and may favor muscle based decoders. Ultimately, further systems level brain-controlled FES experiments could focus on dynamic motions to answer these equations and continue advancing the state of the art for neuroprostheses.

## Ethics statement

This study was carried out in accordance with the recommendations of Guide for the Care and Use of Animals, Office of Laboratory Animal Welfare and the United States Department of Agriculture Animal and Plant Health Inspection Service. The animal care and monitoring protocol was approved by the Institutional Animal Care and Use Committee at the University of Michigan. The study protocol was approved by the Unit for Laboratory Animal Medicine at the University of Michigan.

## Author contributions

AV wrote the manuscript and modified the real-time decoding application to include decoding options necessary to implement ReFIT for different finger groups. ZI developed the original real-time application as well as base code which was modified to apply intention estimation to train ReFIT parameters. SN, PV, CN, and AB assisted with monkey training, fixed issues with the real-time application and experiment set up, and helped develop automated criteria to exclude channels for decoding. MH developed the novel manipulandum with assistance from SN and CN. NN assisted with offline metrics for modulation analysis. PP performed the implant surgery on both subjects and helped shape experiment goals. CC was the principal investigator for this study, providing oversight and advice throughout the entire experiment and writing process.

### Conflict of interest statement

The authors declare that the research was conducted in the absence of any commercial or financial relationships that could be construed as a potential conflict of interest.
