# Supplementary material for: Cortical Decoding of Individual Finger Group Motions Using ReFIT Kalman Filter
Source: Front Neurosci. 2018 Nov 5;12:751. doi: 10.3389/fnins.2018.00751 (PMC6231049; doi:10.3389/fnins.2018.00751)
Supplement: Supplementary file 1 [file Data_Sheet_1.PDF]

Supplementary Material:
Article Title

1 SUPPLEMENTARY TABLES AND FIGURES

1.1 Tables

Finger	Hold Time (ms)	Target Style	Session	Bit Rate (bps)	
				Kalman	ReFIT
Index	750	C-O	1	1.430±0.095	1.982±0.113**
			2	1.438±0.087	2.100±0.103****
		Rand.	1	1.499±0.072	2.217±0.128****
			2	0.845±0.078	1.250±0.112**
		F-E	1	1.641±0.091	2.132±0.076****
			2	0.673±0.052	0.971±0.053****
MRP		C-O	1	1.422±0.081	2.035±0.093****
			2	1.195±0.064	1.653±0.075***
		Rand.	1	1.533±0.0806	1.765±0.073*
			2	1.059±0.088	1.357±0.073*
		F-E	1	1.421±0.052	1.737±0.060***
			2	1.690±0.056	1.926±0.050**

Table S1. Online performance of Monkey W's target acquisition experiments performed with center-out (C-O), random (Rand.), and flex-extend (F-E) target styles. Results measured in bit rate (mean±s.e.m.). Stars indicate a significant improvement of ReFIT over the initial Kalman filter (one-sided two sample t test, $n \approx 150$ trials; with $p < 0.05$ *,$p < 0.001$ **, $p < 1 \times 10^{-4}$ ***, $p < 1 \times 10^{-6}$ ****).

Finger	Hold Time (ms)	Target Style	Session	Bit Rate (bps)	
				Kalman	ReFIT
Index	500	C-O	1	0.836±0.098	1.427±0.090***
			2	0.872±0.077	1.370±0.098***
		Rand.	1	0.930±0.118	1.479±0.122**
			2	1.090±0.130	1.461±0.116*
MRP		C-O	1	1.117±0.073	1.604±0.086***
			2	1.234±0.102	1.457±0.096*
		Rand.	1	1.100±0.098	1.528±0.103*
			2	1.033±0.031	1.468±0.037*

Table S2. Online performance of Monkey N's target acquisition experiments performed with center-out (C-O) and random (Rand.) target styles. Results measured in bit rate (mean±s.e.m.). Stars indicate a significant improvement of ReFIT over the initial Kalman filter (one-sided two sample t test, $n \approx 150$ trials; with $p < 0.05$ *,$p < 0.001$ **, $p < 1 \times 10^{-4}$ ***).

Monkey	Target Style	Hold Time (ms)	Session	Bit Rate (bps)		
				Kalman	ReFIT	
				Manipulandum	Manipulandum	Unrestricted
W	C-O	500	1	1.220±0.066	1.401±0.087*	1.483±0.069
			2	1.457±0.078	1.680±0.090*	$1.836 \pm 0.076^{+}$
N			1	1.301±0.078	1.553±0.085*	1.409±0.077
			2	1.200±0.088	1.430±0.071*	1.279±0.089

Table S3. Online performance of sensory context experiments performed with center-out (C-O) targets. Results measured in bit rate (mean±s.e.m.). Stars indicates a significant improvement of ReFIT over the initial Kalman filter during manipulandum use (one-sided two sample t test, $n \approx 150$ trials; with $p < 0.05$ *,$p < 0.001$ **). A plus indicates a significant performance change between manipulandum use and unrestricted movement while using the ReFIT filter ($p < 0.05$ one-sided two sample t tests, $n \approx 150$ trials).
